# Resistance exercise-induced muscle fatigue is not accompanied by increased phosphorylation of ryanodine receptor 1 at serine 2843

**DOI:** 10.1371/journal.pone.0199307

**Published:** 2018-06-28

**Authors:** Daniel Jacko, Käthe Bersiner, Gerrit Friederichs, Patrick Ritter, Linnea Nirenberg, Jan Eisenbraun, Markus de Marées, Wilhelm Bloch, Sebastian Gehlert

**Affiliations:** 1 Section of Molecular and Cellular Sport Medicine, Institute of Cardiovascular Research and Sport Medicine, German Sport University Cologne, Cologne, Germany; 2 Olympic Base Center Rhineland, Cologne, Germany; 3 Institute of Sport Science, University of Hildesheim, Hildesheim, Germany; 4 Section of Sports Medicine and Sports Nutrition, Faculty of Sport Science, Ruhr University of Bochum, Bochum, Germany; University of Debrecen, HUNGARY

## Abstract

Skeletal muscle fatigue has been shown to be associated with hyperphosphorylation of the ryanodine receptor 1 at serine 2843 (_p_RyR1^Ser2843^), due to chronic overloading exercise. We investigated whether _p_RyR1^Ser2843^, is a mechanism relevant for muscle fatigue also under acute, in contrast to chronic, muscle loading. 24 male subjects (age: 24,8±3,8; height: 182,8±7,2 cm; weight: 82,5±9,9 kg) were evenly (n = 6) assigned to the following four different resistance exercise (RE) groups: hypertrophy- (HYP), strength endurance- (SE), maximum power- (MAX) at the subjects’ 10, 25 and 3 repetition maximum, respectively, and low intensity (LI) RE with 70% of the 10 repetition maximum. Each group completed three different RE volumes (1 set, 5, and 10 sets). Muscle biopsies from the vastus lateralis were taken before and after exercise, analyzed for _p_RyR1^Ser2843^ and examined for association with RE-induced muscle fatigue which was determined as reduction in maximum isometric force (_iso_F^max^) in the quadriceps femoris muscle also before and after exercise.The degree of RE-induced muscle fatigue was specific in terms of set volume as well as of RE mode. _iso_F^max^ was not reduced in any group after one set of RE. Five sets led to a significant reduction of _iso_F^max^ in HYP and SE but not in LI and MAX (p<0,05). Ten sets of RE, as compared to five sets, exclusively induced further muscle fatigue in LI. In terms of RE mode differences, _iso_F^max^ reduction was generally higher in HYP and SE than in MAX and Li after five and ten sets of RE (p<0,05). However, _p_RyR1^Ser2843^ did not show any significant regulation, regardless of exercise condition. We conclude that despite its relevance in reducing muscle contractility in chronic overloading, _p_RyR1^Ser2843^ does not reflect the degree of muscle fatigue exerted by acute hypertrophy-, strength endurance-, maximum power and low intensity-oriented exercise.

## Introduction

Strenuous skeletal muscle activation leads to a reduction of contractile function, thus muscle fatigue. In competitive sports, the loss of physical work capacity due to muscle fatigue decides on victory or defeat, while in sarcopenia but also normal aging, premature muscle fatigue decides on independence and quality of life. Understanding the underlying molecular causes and mechanisms is of great importance for creating strategies for optimized physical training or medication to improve muscle fatigue resistance and maintain skeletal muscle contractility.

A disorder of cytosolic calcium (Ca^2+^) homeostasis in skeletal muscle has already been emphasized in numerous works as a potential cause of reduced contractility [1–4]. The ryanodine receptor type 1 (RyR1) is the main Ca^2+^-channel in skeletal muscle, responsible for a finely tuned release of Ca^2+^ from the sarcoplasmic reticulum (SR) into the cytosol, thereby enabling muscle contraction [5].

Over the last years, some studies highlighted the significance of posttranslational modifications of RyR1 for exercise-induced muscle fatigue [6–10]. The RyR1 is a macromolecular complex consisting of four monomers each about 565 kDa and numerous modulating sites [11]. The phosphorylation of RyR1 monomers has been discussed as a main activity-regulating mechanism [12,13], especially at its serine residue 2843 (_p_RyR1^Ser2843^) [6,8–10]. On one hand, _p_RyR1^Ser2843^ is a physiologic mechanism necessary for an increase in Ca^2+^ release and therefore for inotropy [7]. On the other hand, hyperphosphorylation of RyR1 (meaning that all four monomers of RyR1 are phosphorylated at serine 2843 [9]) has been discussed to be a possible cause for disturbed Ca^2+^ cycling and impaired skeletal muscle contractility [6,9,10].

It has been determined that skeletal muscle of mice [10] as well as humans [6], suffering from heart failure, display hyper _p_RyR1^Ser2843^ which is associated with impaired Ca^2+^ release and premature muscle fatigue. A profound remodeling of the RyR1, inter alia by _p_RyR1^Ser2843^, was detected also in wild type mice in response to chronic high intensity endurance exercise, by Bellinger et al. [9] who also showed that this remodeling was accompanied by a loss of muscle strength. In human skeletal muscle, chronic high intensity endurance exercise [9] as well as acute maximal eccentric resistance exercise [14] were associated with a transient _p_RyR1^Ser2843^, however without available measure of potential loss of muscle function.

Skeletal muscle fatigue is a consequence of various modes of resistance exercises. But to date, no data are available concerning the potential role of _p_RyR1^Ser2843^ in muscle fatigue due to acute practice-oriented exercise regimes. Therefore, a model of four distinct resistance exercise (RE) modes (hypertrophy- [HYP] oriented RE with 10 repetition maximum [RM]; strength endurance- [SE] oriented RE with 25 RM; maximum power- [MAX] oriented RE with 3 RM and low intensity- [LI] oriented RE with 70% of the 10 RM) each with three different volumes (1 set [I], 5 [V] and 10 sets[X], in total 12 distinct load conditions) was used to apply a variety of different workloads on human skeletal muscle. Muscle fatigue was assessed as reduction in maximum isometric force (_iso_F^max^) in *m*. *quadriceps femoris* 25 min post RE loading (F-25post) as compared to baseline (F-pre). Muscle biopsies were also taken before and 25 min post exercise from the vastus lateralis for analysis of _p_RyR1^Ser2843^ via western blot (WB) and immunohistochemistry (IHC).

We hypothesized that RE loading induces a remarkable loss in contractile function post exercise in dependency of the applied exercise mode and set volume. If _p_RyR1^Ser2843^ plays a role for muscle fatigue in one of these conditions, an increase in muscle fatigue should be accompanied by a rise in _p_RyR1^Ser2843^.

## Methods

### Subjects

24 male volunteers (age: 24,8±3,8; height: 182,8±7,2 cm; weight: 82,5±9,9 kg) randomly assigned to one of four groups (n = 6, no statistical differences in age, height and weight across groups) participated in the present study. Subjects were healthy and active but not exceptionally resistance or endurance trained. The protocols used in this study were approved by the ethics committee of the German Sport University of Cologne and align with the Declaration of Helsinki. All subjects were informed orally and in writing of the study’s purpose and possible risks involved before providing written informed consent to participate.

### Standardization of diet and activity before intervention

Participants were instructed to renounce from strenuous activities at least two days prior to the tests. After overnight fasting, subjects were advised to take a standardized meal (Fresubin^®^ Protein Drink, containing 20g Protein, 24,8g carbohydrates, 13,4g fat and in total 1260kJ) two hours before the resting biopsies and three hours before the post-exercise biopsies, respectively. Subjects were allowed to drink water ad libitum.

### Experimental groups

The four experimental groups were chosen to correspond to realistic loading situations relevant for sport practice without aiming to specifically damage skeletal muscle.

**Fig 1:**
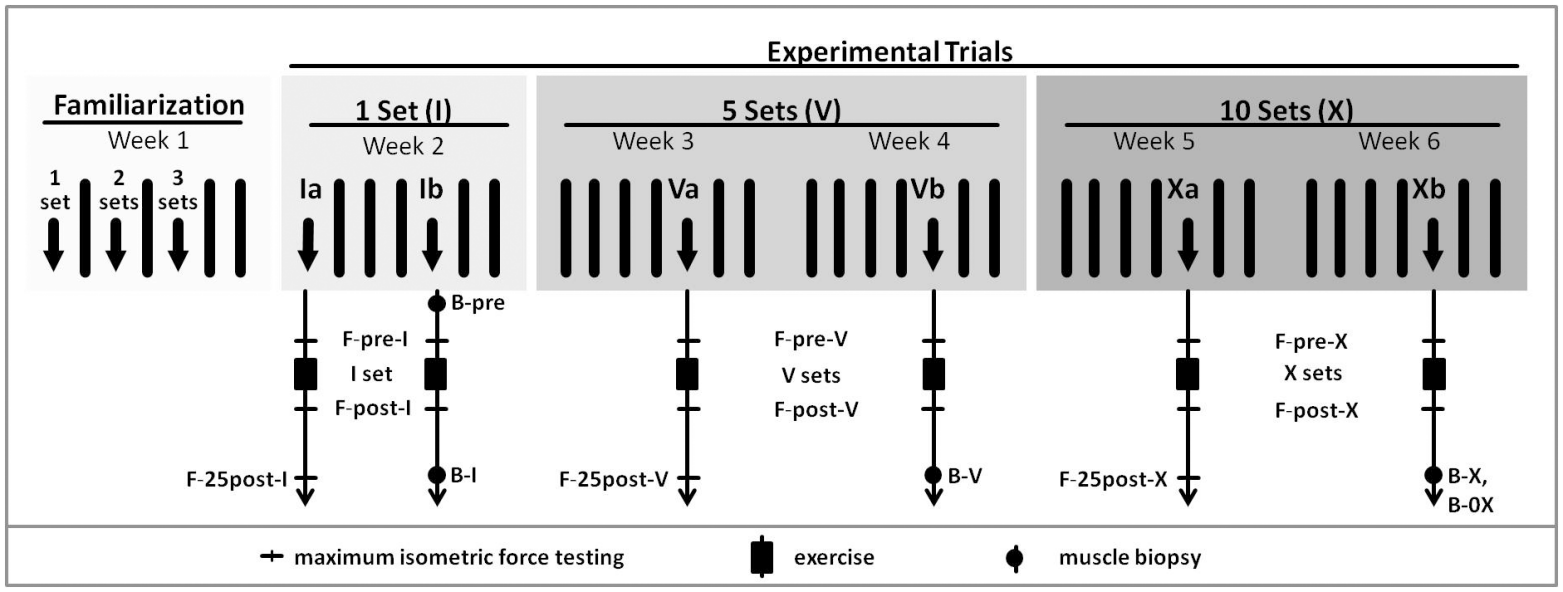
Study design. F-pre-I, -V, -X = maximum isometric force testing 3 min prior to one, five and ten set exercise; F-post-I, -V, -X = maximum isometric force testing 2 min after one, five and ten set exercise; F-25post-I, -V, -X = maximum isometric force testing 25 min after one, five and ten set exercise; B-0 = resting muscle biopsy (non exercising leg); B-I, -V, -X = muscle biopsy 25 min after one, five and ten exercise sets; B-0X = muscle biopsy after ten sets at non exercising leg.

The groups completed either a HYP-, SE-, MAX- oriented RE at the subjects’ 10 (HYP), 25 (SE) and 3 (MAX) RM or a LI, health sports-oriented RE with 70% of their 10 RM. Each group completed three different RE volumes (I set, V sets and X sets) on separate days, over a time period of all in all five weeks (Fig 1).

In order to meet the requirements of RM in each set, the resistance was adjusted in the subsequent set, if subject were not able to complete their demanded amount of repetitions in the previous set.

### Exercise modalities

The rest interval between each set for all groups was 2 min and the time under tension for each repetition was 6 s with a contraction pattern of 2 s concentric, 1 s isometric, 2 s eccentric and 1 s isometric. The contraction pattern was maintained by a visual biofeedback system. The exercises were performed one legged on a leg extension machine (CORE80, Gym80, Gelsenkirchen, Germany) which was retrofitted with a range sensor (SX80-1000-5R-SA, Waycon, Munich, Germany) and a force sensor (S-Beam, KM1506 K 5KN 0000, Megatron, Munich Germany). Before the experimental trial, a familiarization phase was carried out during which individual training load was determined as well as subjects became acquainted with the training device and the strength testing procedure. While testing, subjects were encouraged vocally.

Muscle fatigue was assessed as _iso_F^max^ reduction between baseline (F-pre) and 25 min after exercise (F-25post).

### Study design

To study muscle fatigue-related processes based on muscle biopsies, muscle tissue has to be obtained at the same time point as magnitude of functional impairment is assessed. This is associated with organizational challenges. On that account, every volume/trial (I, V and X) in each group was carried out on two days, “a” and “b” (Ia & Ib, Va & Vb, Xa & Xb), respectively (Fig 1). The a-days, hereafter also termed fatigue days, served the assessment of the load-induced muscle fatigue. The b-days served the extraction of muscle biopsies. The procedures described below followed the exact same plan on day a as on day b: the subjects’ _iso_F^max^ were determined in rested, non-fatigued state (F-pre) after a standardized warm up. Subsequently, subjects carried out their loading protocol. After completion, a second _iso_F^max^-test was carried out to determine the immediate effect of loading-induced muscle fatigue (F-post). Thereby, F-pre and F-post from days a and b were used to assess reproducibility of the muscle fatigue assessment between both days. The only difference in procedures between days a and b was that on day a, a further _iso_F^max^-test was performed 25 min after loading (F-25post), whereas on day b, a muscle biopsy was taken 25 min after loading from the exercised leg.

Consequently, provided that F-pre and F-post on days a and b are equivalent and reliable, it can be assumed that muscle fatigue assessed 25 min post exercise on a-days is valid also for b days. In this case, the contractile impairment, assessed by F-25post on a-days, can serve as benchmark of functional status also for b-days, allowing to study the relationship between muscle fatigue and ongoing molecular processes at the same time point.

### Familiarization

Familiarization started one week prior to the main experimental trial. In this phase, subjects reported to the laboratory three times, separated by one day. On the first day, exercise and control leg were chosen randomly. For warm up, subjects cycled on an ergometer for 5 min with a load of 1,5 watts/kg bodyweight. After a 2 min rest, subjects completed one set of 10 repetitions with a load of one fourth of their body weight. After a rest of 3 min, two _iso_F^max^ tests, separated by 1 min, were completed. From the _iso_F^max^ data, the individual, group specific exercise weight was estimated and after a rest of 3 min, subjects starter their exercise. 2 min after completion, an immediate _iso_F^max^ (F-post) was carried out. After a pause of 25 min during which subjects stayed seated on the training device, another two _iso_F^max^ tests (F-25post), again separated by 1 min, were completed. The procedures of the other two familiarization days followed the exact same plan as on the first day, however with two and three sets, respectively.

### Experimental trial

In the following week, after familiarization, the first experimental trial started with the fatigue day by means of I set (Ia). Subjects reported to the laboratory between 8 a.m. and 10 a.m. The procedure was exactly the same as on familiarization days (warm up → F-pre → RE → F-post → F-25post). After a pause of 3 days, the first biopsy day (Ib) was carried out which differed only in that F-25post was replaced by a muscle biopsy (B-I). Moreover, a resting biopsy (B-0) was taken from the non exercised leg one hour prior to the exercise.

After a pause of six days, during which the muscle recovered from the strain and biopsy, the V set trial begun with the fatiguing measure day (Va) followed by the biopsy day (Vb) with one biopsy (B-V) after completion of the exercise. To ensure full recovery with increasing number of sets, the duration of the pause was extended from three to six days between a- and b-days. Again, after six days pause, finally the X set trial started with Xa, followed after a week by Xb. On day Xb, two biopsies were taken; one by default from the strained leg 25 min after the exercise (B-X) and a second one immediately after B-X from the rested leg (B-0X) whereby B-0X was for obtaining possible cross over effects.

### _iso_F^max^ testing

Subjects sat upright in the leg extension device (CORE80, Gym80, Gelsenkirchen, Germany). The lever arm of the device was set at 120° knee angle and mechanically locked, so that subjects could perform maximum isometric quadriceps contraction. Prior to each test trial, the force sensor was reset before subjects placed their distal shin on the pad of the lever arm. Subjects were instructed to apply an initial force of 30–50 Nm to the pad and exert their _iso_F^max^ when feeling ready. They were supposed to execute explosively and maintain _iso_F^max^ for 4 s. When the applied force exceeded 100 Nm, a countdown started, giving the subjects an acoustic signal after 4 s that contraction time ended. Subjects were allowed to hold the grips at the right and left beside the seat in order to prevent the hip from lifting off the seat.

### Biopsies and tissue processing

A total of five muscle biopsies were taken from each subject: three post-exercise biopsies from the loaded leg (B-I, B-V, B-X) and two from the resting leg as baseline reference (B-0, B-0X). All biopsies were obtained from the middle portion of the vastus lateralis between the spina iliaca anterior superior and the lateral part of the patella. Immediately after completion of the F-25post test, a local anesthetic injection was set. A 2 cm incision was made through skin and fascia and tissue was gathered 2,5 cm below the fascia using the percutaneous needle biopsy technique [15].

Muscle samples were freed from blood and non muscle material, embedded in Tissue Tek (Sakura Finetek, Zoeterwoude, Netherlands), frozen in liquid nitrogen-cooled isopentane and stored at -80°C for further analysis.

### Immunohistochemistry

7 μm cross-sectional slices were obtained from the frozen muscle tissue using a cryo-microtome Leica CM 3050 S (Leica Microsystems, Nußbach, Germany) and placed on Polysine^™^ microscope slides (VWR International, Leuven, Belgium). Sections were fixed for 8 min in -20°C pre-cooled acetone and air dried for 15 min at room temperature (RT), before blocking for one hour at RT with TBS (tris buffered saline, 150mM NaCl, 10 mM Tris-HCl, pH 7,6) containing 5% BSA (bovine serum albumin). After blocking, sections were incubated over night with rabbit polyclonal primary antibody which detects phosphorylated RyR1 (host: rabbit; ab59225, Abcam, Cambridge, UK), diluted 1:150 in 0,8% BSA. To confirm antibody specificity, control sections were incubated in TBS containing 0,8% BSA but without primary antibody. After incubation, sections were washed 3 times with TBS and incubated for one hour with biotinylated goat anti rabbit secondary antibody (Dako, Glostrup, Denmark), diluted 1:400 in TBS, at RT. Thereafter, sections were washed 3 times before incubation for one hour at RT with streptavidin biotinylated horseradish peroxidase complex (Amersham Biosciences, Uppsala, Sweden) 1:400 in TBS. Sections were washed once again and immunohistochemical staining was finalized using a 3,3’-diaminobenzidine (DAB) solution (0,09 M phosphate buffer, pH 7,4; 2,2 mM DAB; 7,03 mM ammonium chloride; 0,93 mM nickel sulfate; 10,44 mM β-D-glucose and 0,024 μM glucose oxidase).

For fiber type specific differentiation of RyR1 phosphorylation pattern, consecutively cut sections were incubated with A4.951, 1:400 (DSHB, Iowa, USA) which reliably detects type 1 muscle fibers, and stained according to the previously described procedure.

All sections of one subject were mounted on one slide, respectively, and all slides were stained within a single batch using the same antibody dilution and development time to minimize variability in staining efficiency. After dehydration, the stained sections were embedded in Entellan (Merck, Darmstadt, Germany) and supplied with a cover slip.

Images from the stained sections were captured using a camera (AxioCam MRm, Software: Axiovision Rel. 4.6, Zeiss GmbH, Jena, Germany) coupled confocal light microscope (Axiovert 200 M., Zeiss GmbH, Jena, Germany). The background intensity was standardized, set separately for each slide. Type 1 and 2 fibers were identified by comparing _p_RyR1 stained sections with consecutive sections stained with A4.951. Fiber type specific phosphorylation pattern was quantified in the sarcoplasmic region of fibers via optical densitometry using the software ImageJ^®^ (National Instruments of Health, USA). Intracellular _p_RyR1^Ser2843^ was set as mean staining intensity.

### Western blot

Tissue was homogenized in ice-cold lysis buffer (Cell Signaling, Boston, USA) supplemented with a protease and phosphatase inhibitor cocktail (Halt^™^, Thermo Scientific, Waltham, USA), using a micro-dismembrator (Braun, Melsungen, Germany). The protein concentration of each sample was quantified with a Lowry test kit (Bio Rad, Munich, Germany) and a multiplate reader (Multiskan FC, Thermo Scientific, Waltham, USA). For gel electrophoresis, samples were added with 3x Laemmli buffer and heated at 95°C for 3 min. Samples were cooled down to room temperature and loaded in a precast 6–12% bis-tris polyacrylamid gel system (Criterion^™^ XT, Bio Rad, Munich, Germnay). After electrophoretic separation (100 volt, constant) in XT MOPS Running Buffer (Bio Rad, Munich, Germany), proteins were transferred (1,2 amp, 25 volt max, 34 minutes) to a polyvinylidene difluoride (PVDF) membrane (Bio Rad, Munich, Germany) using a semi dry blotting system (Criterion^™^, Bio-Rad, Munich, Germany). The membranes were blocked in 5% non-fat dry milk for one hour at RT and incubated over night at 4°C with phospho RyR1 specific antibody (same as described in immunohistochemical staining section, host: rabbit, diluted 1:2000 in 5% BSA, ab59225, Abcam, Cambridge, UK). Membranes were washed 3 times with TBST (TBS added with 1% Tween^®^20 [Sigma Aldrich, St. Louis, USA]) and afterwards incubated for one hour at RT with the secondary antibody (goat anti rabbit, diluted 1:10.000 in 5% non-fat dry milk, ThermoScientific, Rockford, USA) and then washed in TBST. Proteins were detected by an enhanced chemo-luminescence assay (ECL-Kit, Amersham Life Science, Buckinghamshire, UK) exposed to an X-ray film (Kodak, X-OMAT Engineering, Eastman Kodak Co., Rochster, NY).

Membranes were stripped with a commercially available stripping buffer (ThermoScientific, Rockford, USA) washed with TBST and re-incubated in the same way as described above, with primary monoclonal antibody against _total_RyR1 (host: mouse, diluted 1:5000 in 5% BSA, ab2868, Abcam, Cambridge, UK) and secondary goat anti mouse antibody (diluted 1:10.000 in 5% non-fat dry milk, ThermoScientific, Rockford, USA).

### Statistics

For retest reliability measures, previously nonparametric Wilcoxon test for related samples (p<0,05) was carried out before ICC (interclass correlation coefficient, model: alpha, two way mixed, type: absolute agreement, cin 95%) was calculated. To asses load volume-induced differences in muscle fatigue as well as differences in _p_RyR1^Ser2843^ and _total_RyR1 within groups, nonparametric Friedman’s ANOVA for related samples (p<0,05) was used with Benjamini Hochberg correction for multiple comparisons. To examine differences across groups, data were transformed to relative values based on pre conditions and nonparametric Kruskal-Wallis one way ANOVA for independent samples (p<0,05) with Benjamini & Hochberg correction for multiple comparisons was carried out.

## Results

### Effect of resistance exercise on muscle fatigue

Test-retest reliability measures showed a very good reproducibility for F-pre and F-post between fatigue days (Ia, Va, Xa) and biopsy days (Ib, Vb, Xb) (data not shown), allowing to derive relationships between F-25post values and biopsies taken 25 min post exercise.

**Fig 2:**
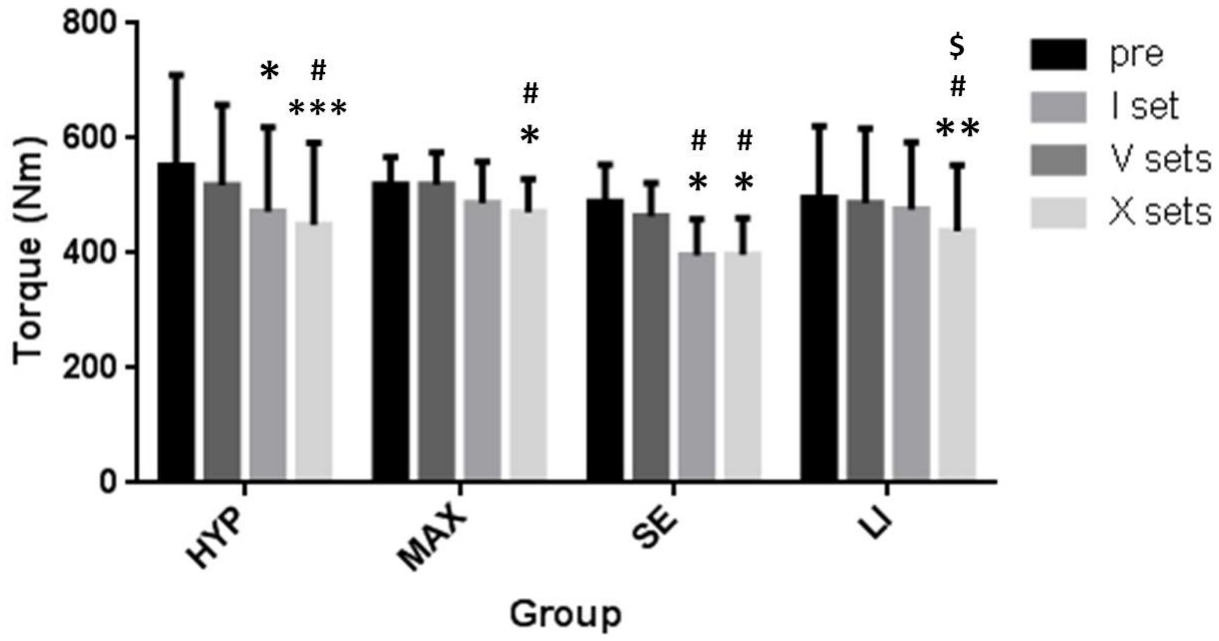
Reduction in voluntary isometric torque in dependency of previously administered resistance exercise (RE) load volume. Maximal voluntary isometric torque of quadriceps femoris was assessed before (pre) and 25 min after completion of one set (I), five (V) and ten (X) sets of resistance exercise in the following groups: hypertrophy (HYP), strength endurance (SE), maximum power (MAX) at the subjects’ 10, 25 and 3 repetition maximum (RM), respectively, and low intensity (LI) oriented RE with 70% of the subjects’ 10 RM. * = different to pre with p<0,05, ** p<0,01, *** p<0,001; # = different to I; $ = different to V; error bars are 1*SD of the mean.

Within groups, different load volumes led to distinct _iso_F^max^ reductions (Fig 2). In the HYP group, the _iso_F^max^ decreased significantly after V sets compared to pre (p<0,05) as well as after X sets compared to pre (p<0,001) and I set (p<0,05). In the MAX group, only X sets led to an _iso_F^max^ reduction which was significant compared to pre (p<0,05) and also I set (p<0,05). In the SE group, both, V and X sets, resulted in significant _iso_F^max^ loss compared to pre (p<0,05) and I set (p<0,05). In the LI group, exclusively the completion of X sets led to a reduction of the _iso_F^max^ which was significant compared to pre (p<0,01), I (p<0,05) and V (p<0,05).

**Fig 3:**
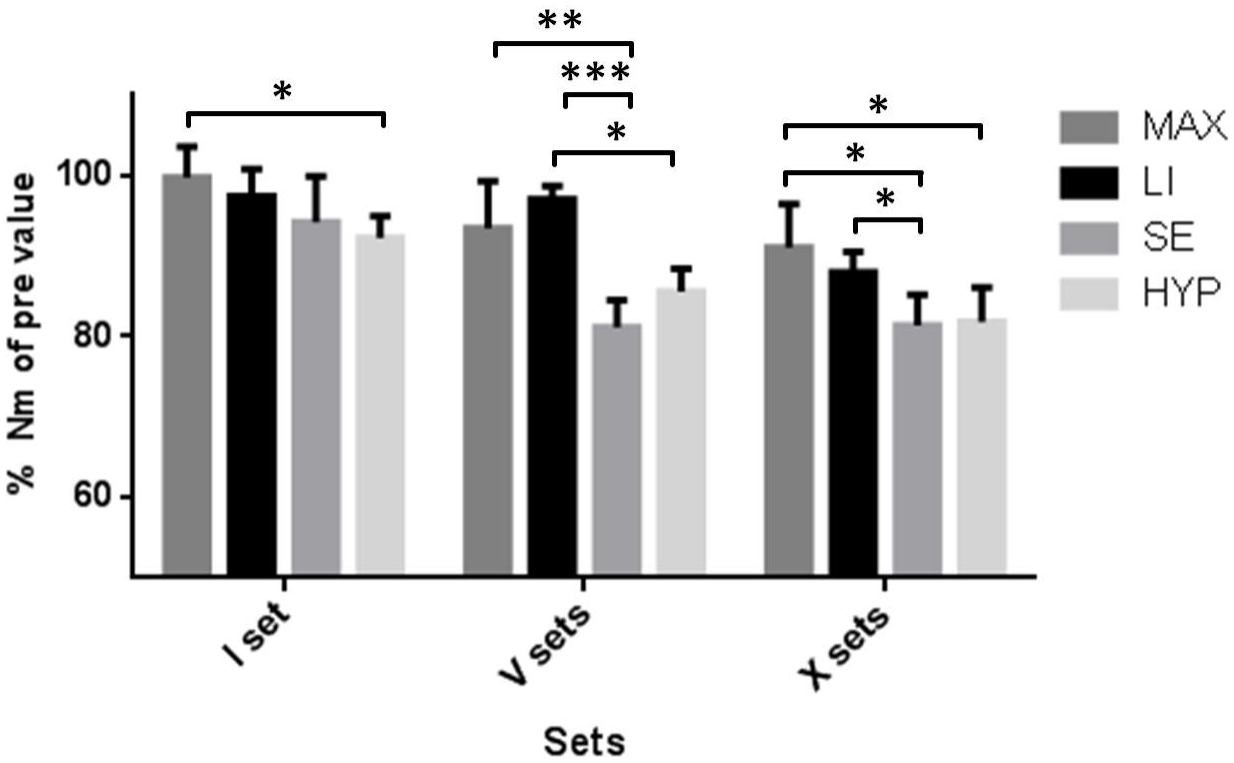
Comparison of the reduction of the maximal isometric torque after one set (I), five (V) and ten sets (X) of exercise across groups. Maximal voluntary isometric torque of quadriceps femoris was assessed before (pre) and 25 min after completion of one set (I), five (V) and ten (X) sets of resistance exercise in the following groups: hypertrophy (HYP), strength endurance (SE), maximum power (MAX) at the subjects’ 10, 25 and 3 repetition maximum (RM), respectively, and low intensity (LI) oriented RE with 70% of the subjects’ 10 RM. Percentages are based on individual pre values. Horizontal lines indicate differences between resistance exercise groups. * = p<0,05; ** = p<0,01; *** = p<0,001; error bars are 1*SD of the mean.

Comparison of muscle fatigue due to RE set volume across groups shows that there is no difference between HYP and SE as well as between MAX and LI. However, there are distinct differences between HYP/SE as compared to MAX/LI (Fig 3). In detail, I set of HYP load has a significantly higher effect on muscle fatigue than I set MAX load (p<0,05). V sets of SE (p<0,01) as well as HYP load (p<0,001) lead to significantly greater _iso_F^max^ loss than V sets of LI. Furthermore, _iso_F^max^ decline is more pronounced after V sets SE than V sets MAX (p<0,05). X sets SE result in greater muscle fatigue than X sets LI (p<0,05) and MAX (p<0,05). Also, the magnitude of muscle fatigue is more pronounced due to HYP than to MAX (p<0,05).

### Determination of resistance exercise-induced _p_RyR1^Ser2843^

**Fig 4:**
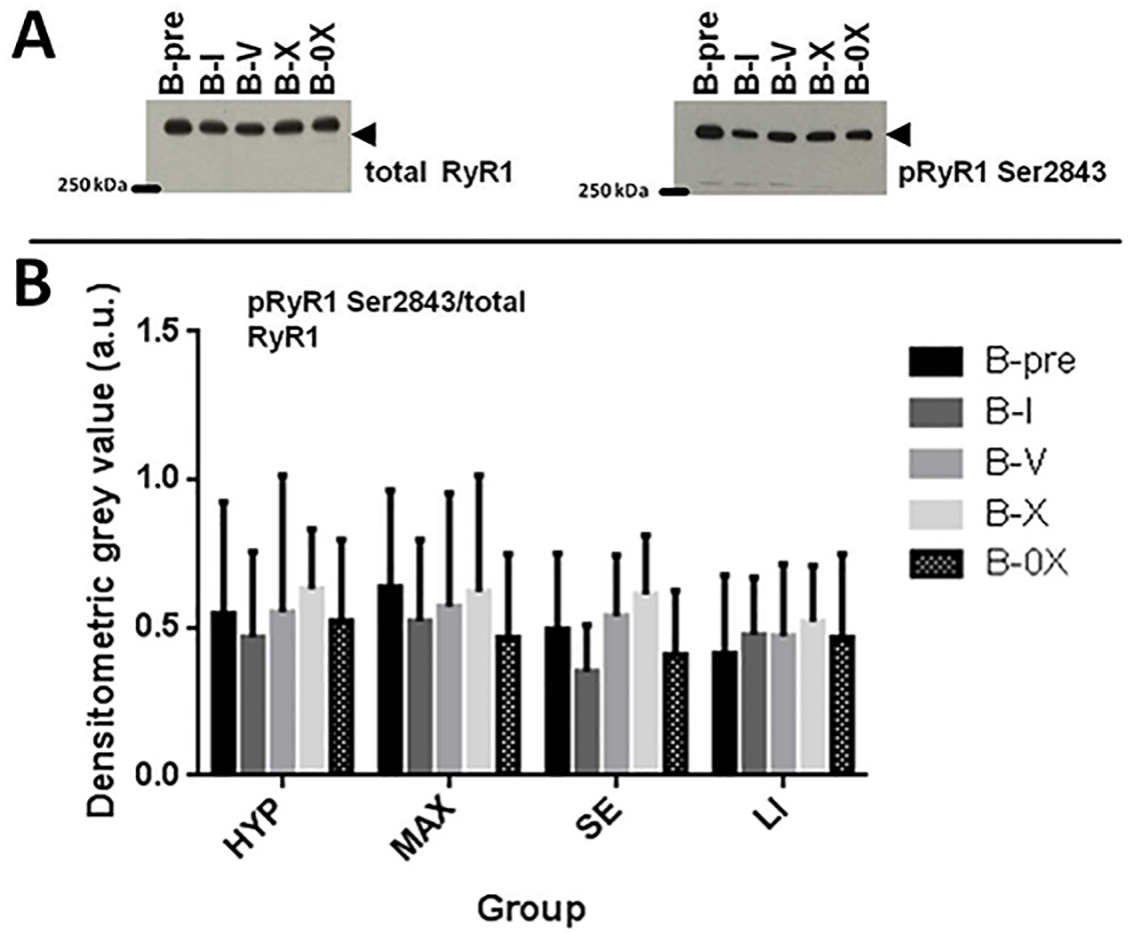
Western blotting analysis of RyR1 phosphorylation at serine 2843 (_p_RyR1^Ser2843^) in human skeletal muscle tissue. **A**: Representative western blots of _p_RyR1^Ser2843^ (left) and _total_RyR1 (right). Tissue was analyzed from muscle biopsies taken prior to exercise (B-pre), 25 min after completion of one set (B-I), five (B-V) and ten sets (B-X) from vastus lateralis of the loaded leg as well as 25–30 min after X set exercise from vastus lateralis of the non-exercised leg (B-0X). _Total_RyR1 was used as loading control. **B**: Bar graph of _p_RyR1^Ser2843^/_total_RyR1. a.u. = arbitrary units. Error bars are 1*SD of the mean.

**Fig 5:**
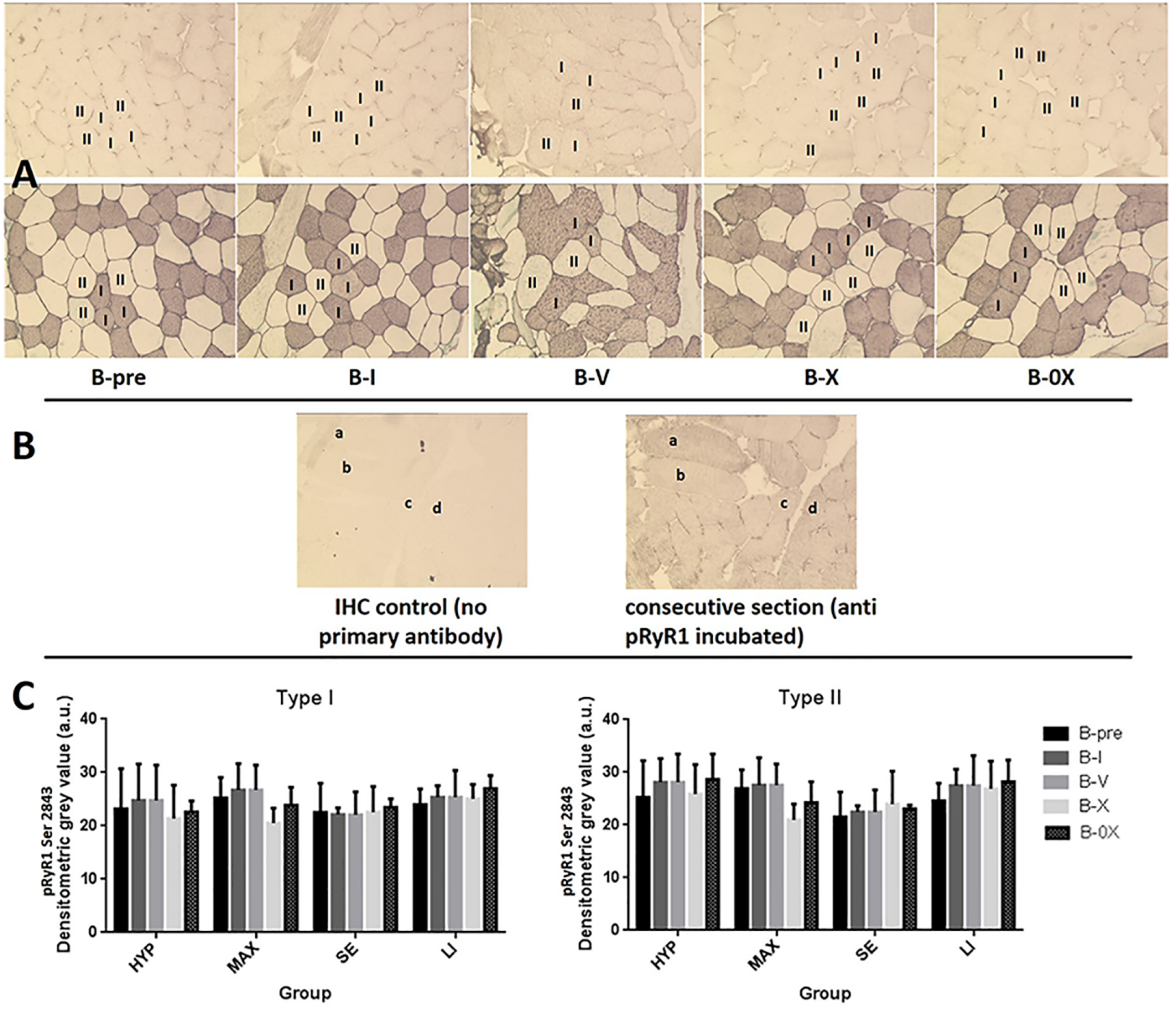
Immunohistochemistry: RyR1 phosphorylation at serine 2843 (_p_RyR1^Ser2843^) in type I and type II human skeletal muscle fibers. **A**: Representative immunohistochemistry. At the top: _p_RyR1^Ser2843^; below: fiber typing. Dark staining displays type 1 myofibers, unstained displays type 2 myofibers (MHC I = myosin heavy chain I). The _p_RyR1^Ser2843^ was analyzed from muscle biopsies taken prior to exercise (B-pre), 25 min after completion of one set (B-I), five (B-V) and ten sets (B-X) from vastus lateralis of the loaded leg as well as 25–30 min after X set exercise from vastus lateralis of the non-exercised leg (B-0X). **B**: Immunohistochemical control. At the left: section where no primary antibody was used; at the right: consecutive section incubated with anti _p_RyR1^Ser2843^. **C**: Bar graph, _p_RyR1^Ser2843^. a.u. = arbitrary units. Error bars are 1*SD of the mean.

In order to determine whether RE leads to an increase in _p_RyR1^Ser2843^, we performed western blot and immunohistochemical analysis from muscle biopsies taken prior to and 25 min post exercise from vastus lateralis. The _p_RyR1^Ser2843^ does not show any significant regulation in any group at any time point. This applies to both, the WB (Fig 4) as well as the fiber type specific IHC (Fig 5). There is also no correlation between exercise-induced muscle fatigue and _p_RyR1^Ser2843^ due to WB or IHC analysis (data not shown).

## Discussion

Previous work has shown that excessive _p_RyR1^Ser2843^ due to heart failure-associated chronic hyperadrenergic state [6,9,10] as well as chronic strenuous endurance exercise [9] is a potential cause for reduced muscle contractility. We aimed to determine whether _p_RyR1^Ser2843^ is also associated with muscle fatigue that is induced by variations in loading of acute resistance exercise programs, in contrast to the above mentioned chronic situations.

Our main findings are that resistance exercise led to a pronounced muscle fatigue which in magnitude depends on RE volume and mode (Figs 2 and 3). However, there was no significant regulation in _p_RyR1^Ser2843^ at any time point (Figs 4 and 5) and therefore, no correlation between _p_RyR1^Ser2843^ and muscle fatigue was detected (data not shown).

Previously, Reiken et al. [10] postulated a mechanism by which RyR1 becomes phosphorylated due to a chronic adrenergic state via the epinephrine/AC (adenylyle cyclase)/cAMP (cyclic adenosine mono phosphate)/PKA (protein kinase A) axis [6,8]. Bellinger et al. [9] simulated a chronic adrenergic stimulation in wild type mice, exposing them to several weeks of excessive strenuous exercise. They were able to show a direct link between chronic overloading, _p_RyR1^Ser2843^ levels and loss in contractile function. However, in this study it was not examined whether repetitive exercise was accompanied by chronic adrenergic state. The results at least indicate that an accumulation of long term physical stress is a main stimulus leading to increased _p_RyR1^Ser2843^, whereby _p_RyR1^Ser2843^ is responsible for muscle fatigue.

In the present study, RE modes relevant for practice were applied. The loads and biopsy time points were chosen to induce and determine an acute muscle fatigue, in clear contrast to chronic overloading. Referring to the epinephrine dependant _p_RyR1^Ser2843^, it is well known that also acute resistance exercise leads to an increase in venous blood epinephrine concentration [16–18]. However, this increase is highly transient and resting values are restored after 15 min post exercise at the most [19,20]. It might thus be speculated that this burst of adrenal stimulation of skeletal muscle β2-receptors due to acute exercise might be too short in duration to induce a sustained increase in _p_RyR1^Ser2843^, explaining why no _p_RyR1^Ser2843^ regulation could be observed in any exercise mode in the present study (Figs 4 and 5).

On the other hand, the absence of any increase in _p_RyR1^Ser2843^ due to resistance exercise contradicts previous findings of Gehlert et al. [14] but could be explained by the diversity of exercise modalities used in their and in our study. The most striking difference is the contraction pattern. Gehlert et al. [14] used three sets of eight repetitions of isokinetic eccentric contractions (ECC) with maximum effort, without any familiarization phase. It is well known that this kind of exercise has a much higher potential to cause profound muscle damage than the regular exercise used in the present study, i.e. exercise consisting of concentric, isometric and ECC phases as well as an additional foregoing familiarization [21,22]. The ECC-induced structural damage [21] as well as myofibrillar stretch [23,24] have been shown to increase intracellular calcium concentrations [Ca^2+^]_i_ post exercise and these elevations are more pronounced in ECC than in isometric [24] or concentric [25] contraction patterns. [Ca^2+^]_i_ is an activator of Ca^2+^/calmodulin-dependent protein kinase 2 (CamKII) which in turn has been shown to affect RyR1 phosphorylation [13,26]. This might explain how RE could lead to a sustained increase in _p_RyR1^Ser2843^ in an epinephrine/PKA axis independent manner and why eccentrically—but not regular, i.e. concentric-isometric-eccentric-loaded muscles show a _p_RyR1^Ser2843^ increase post exercise.

Furthermore, it is to note that the RyR1 is a macro molecular complex of about 2200 kDa which can be modulated in several ways at a multitude of modification sites [27]. Besides, the phosphorylation at serine 2843, also S-nitrosylation, dissociation of phosphodiesterase 4D3 [9] and oxidation [28,6] have been demonstrated to affect RyR1 Ca^2+^ release and the associated muscle function. Therefore, it should be considered that RyR1 dysfunction might have contributed to muscle fatigue also in the present study due to other modifications mentioned above, however not through _p_RyR1^Ser2843^.

In summary, acute resistance exercise leads to muscle fatigue. The degree of muscle fatigue depends on RE mode as well as volume of loading. However, none of the RE conditions used in the present study was associated with a significant increase in _p_RyR1^Ser2843^.

We conclude that despite its relevance in reducing muscle contractility in chronic adrenergic stimulation, _p_RyR1^Ser2843^ is not associated with muscle fatigue due to acute hypertrophy-, strength endurance-, maximum power and low intensity-oriented resistance exercise with a regular concentric-isometric-eccentric contraction pattern. Therefore, _p_RyR1^Ser2843^ does not serve as a valid and consistent indicator for determining reduced contractile function in response to acute loading in human muscle.

Possibly, _p_RyR1^Ser2843^ could play a more pronounced role in muscle fatigue due to chronic to maximum eccentric exercise associated with increased muscle damage and an increase in [Ca^2+^]_i_.

## Supporting information

S1 Fig Individual representation of muscle fatigue (% of reduction in maximum voluntary isometric torque compared to pre value) associated with RyR1 phosphorylation at serin 2843 in hypertrophy-oriented group. Maximal voluntary isometric torque of quadriceps femoris was assessed before (pre) and 25 min after completion of one set (I), five (V) and ten (X) sets of resistance exercise. Biopsies were taken at the same time points and analyzed via western blot for RyR1 phosphorylation. An additional biopsy was taken after ten sets from the non-loaded leg as negative control.
(TIF)

S2 Fig Individual representation of muscle fatigue (% of reduction in maximum voluntary isometric torque compared to pre value) associated with RyR1 phosphorylation at serin 2843 in maximum power-oriented group. Maximal voluntary isometric torque of quadriceps femoris was assessed before (pre) and 25 min after completion of one set (I), five (V) and ten (X) sets of resistance exercise. Biopsies were taken at the same time points and analyzed via western blot for RyR1 phosphorylation. An additional biopsy was taken after ten sets from the non-loaded leg as negative control.
(TIF)

S3 Fig Individual representation of muscle fatigue (% of reduction in maximum voluntary isometric torque compared to pre value) associated with RyR1 phosphorylation at serin 2843 in strength endurance-oriented group. Maximal voluntary isometric torque of quadriceps femoris was assessed before (pre) and 25 min after completion of one set (I), five (V) and ten (X) sets of resistance exercise. Biopsies were taken at the same time points and analyzed via western blot for RyR1 phosphorylation. An additional biopsy was taken after ten sets from the non-loaded leg as negative control.
(TIF)

S4 Fig Individual representation of muscle fatigue (% of reduction in maximum voluntary isometric torque compared to pre value) associated with RyR1 phosphorylation at serin 2843 in low intensity-oriented group. Maximal voluntary isometric torque of quadriceps femoris was assessed before (pre) and 25 min after completion of one set (I), five (V) and ten (X) sets of resistance exercise. Biopsies were taken at the same time points and analyzed via western blot for RyR1 phosphorylation. An additional biopsy was taken after ten sets from the non-loaded leg as negative control.
(TIF)

S5 Fig Dephosphorylation experiment using alkaline phosphatase (AP) to confirm phospho-specificity of the used antibody. In short: Tissue was prepared as described in the section Methods, Western Blot. Crude cell lysates were incubated with: Line 1: CIP-Buffer, without AP (calf intestinal [CIP]), for testing of possible influence of Buffer itself on pRyR1 and total RyR1 signal. Line 2 and 3: CIP-Buffer without AP, incubated for 30 (line 2) and 60 (line 3)min at 37°C, for testing of possible influence of incubation temperature on pRyR1 and total RyR1 signal. Line 4 and 5: CIP-Buffer with AP added in two different concentrations (recommended → line 4 and lower → line 5). Following incubation with total RyR1 (mouse), a clear signal is visible at predicted band size, which is quite even in intensity across the lines. After stripping the membrane and reincubation with pRyR1 antibody (rabbit), a clear reduction in signal intensity is observable only in the AP added lines 4 and 5. This is a positive indication for us that pRyR1 antibody recognizes differences in phosphorylation and that our results are not based on technical issues.
(TIF)
