# Supplementary figures and images for: Resistance exercise-induced muscle fatigue is not accompanied by increased phosphorylation of ryanodine receptor 1 at serine 2843
Source: PLoS One. 2018 Jun 28;13(6):e0199307. doi: 10.1371/journal.pone.0199307 (PMC6023196; doi:10.1371/journal.pone.0199307)

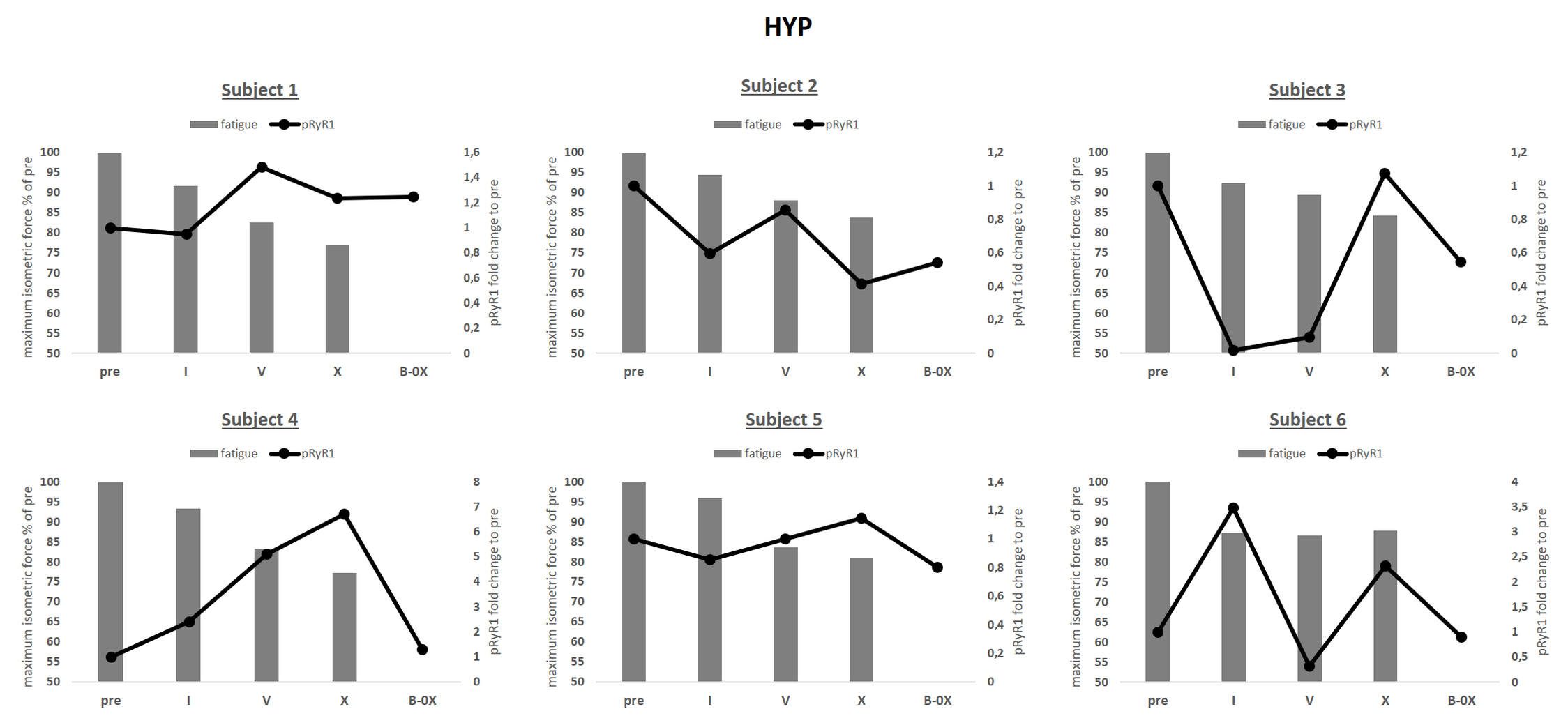

Supplement: S1 Fig — Maximal voluntary isometric torque of quadriceps femoris was assessed before (pre) and 25 min after completion of one set (I), five (V) and ten (X) sets of resistance exercise. Biopsies were taken at the same time points and analyzed via western blot for RyR1 phosphorylation. An additional biopsy was taken after ten sets from the non-loaded leg as negative control. (TIF) [file pone.0199307.s001.tif]

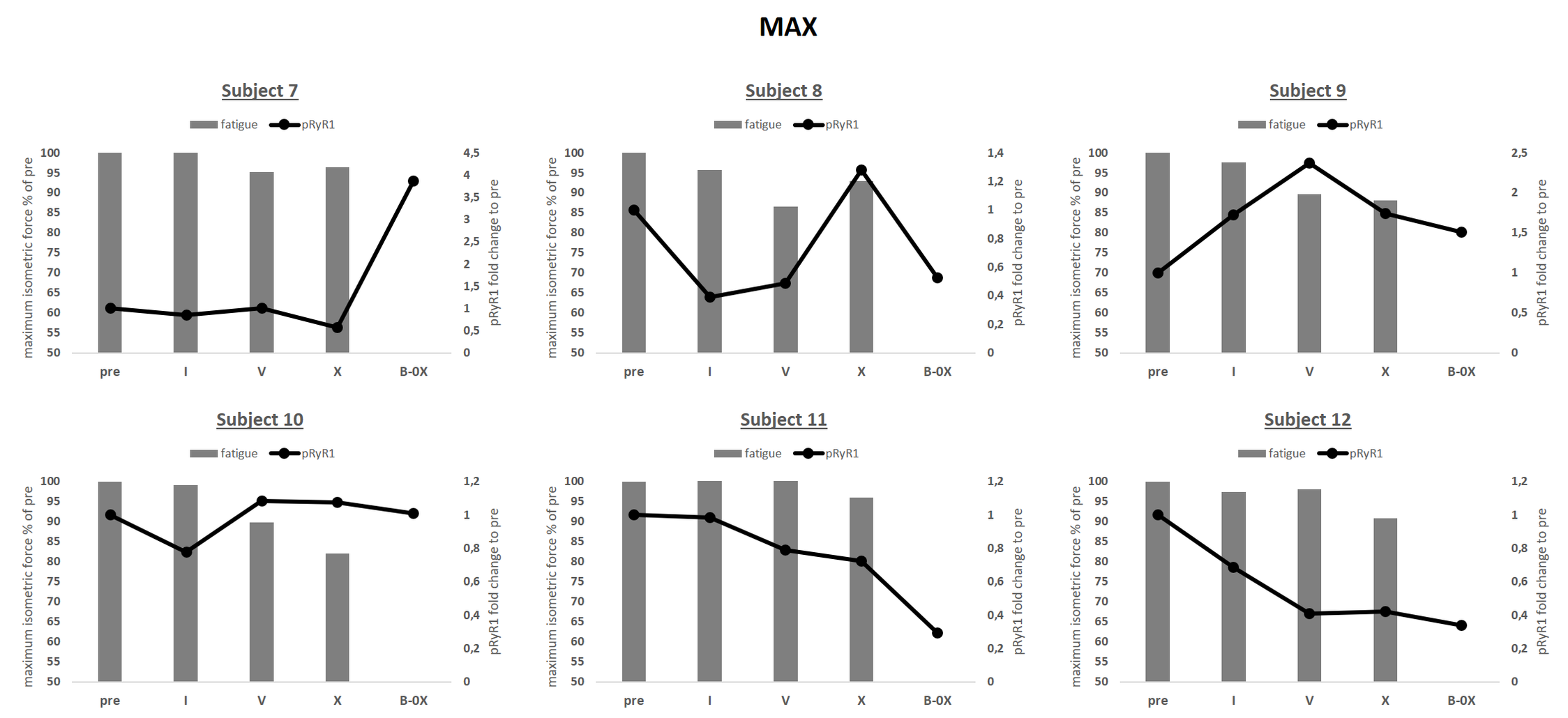

Supplement: S2 Fig — Maximal voluntary isometric torque of quadriceps femoris was assessed before (pre) and 25 min after completion of one set (I), five (V) and ten (X) sets of resistance exercise. Biopsies were taken at the same time points and analyzed via western blot for RyR1 phosphorylation. An additional biopsy was taken after ten sets from the non-loaded leg as negative control. (TIF) [file pone.0199307.s002.tif]

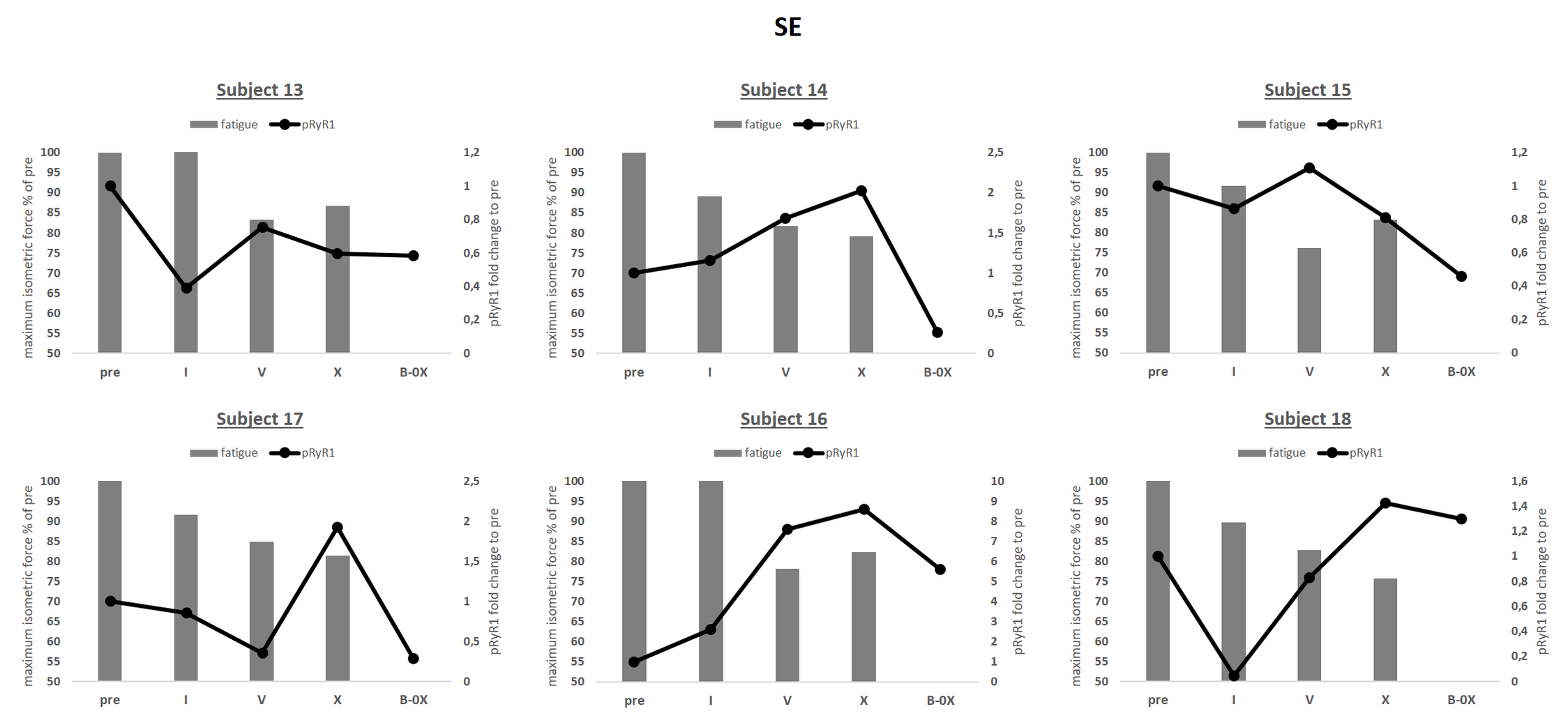

Supplement: S3 Fig — Maximal voluntary isometric torque of quadriceps femoris was assessed before (pre) and 25 min after completion of one set (I), five (V) and ten (X) sets of resistance exercise. Biopsies were taken at the same time points and analyzed via western blot for RyR1 phosphorylation. An additional biopsy was taken after ten sets from the non-loaded leg as negative control. (TIF) [file pone.0199307.s003.tif]

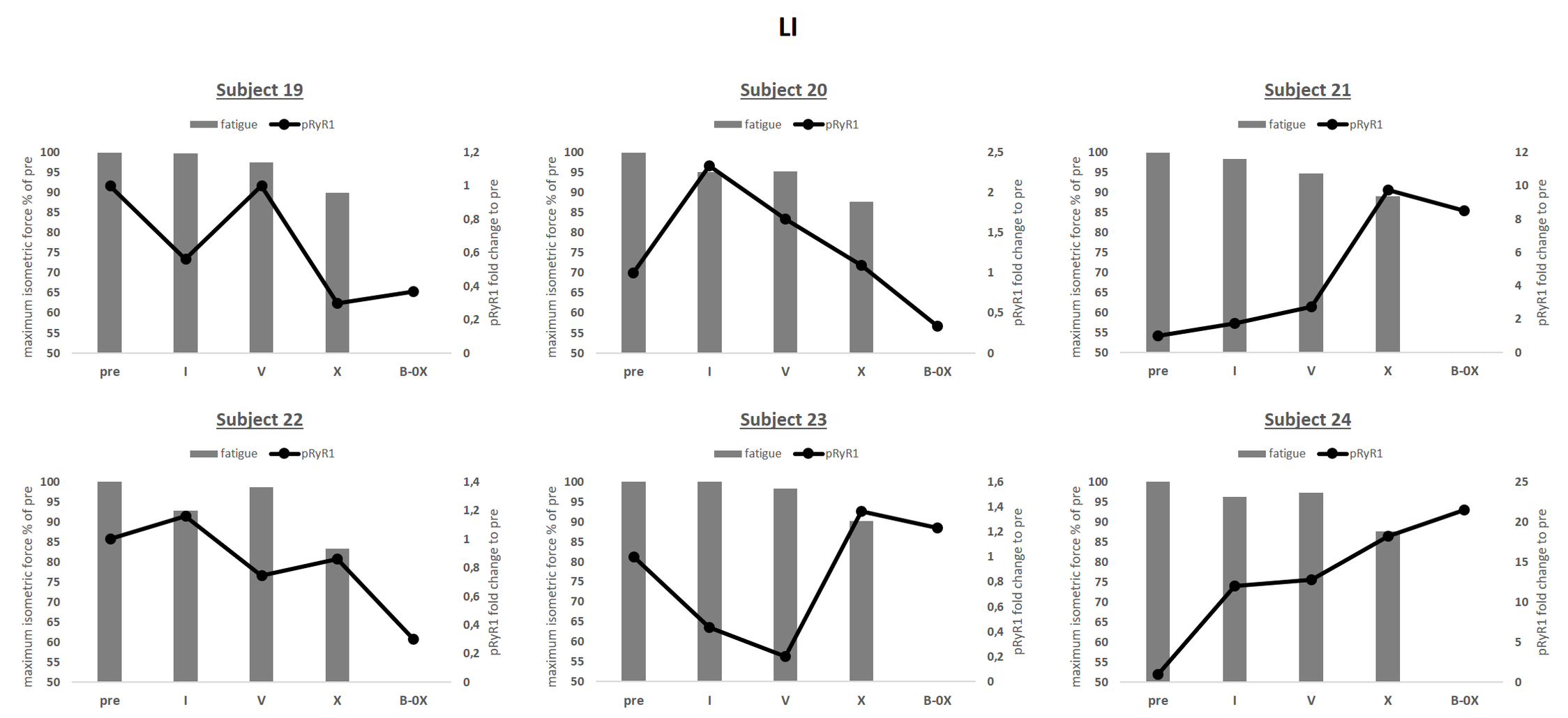

Supplement: S4 Fig — Maximal voluntary isometric torque of quadriceps femoris was assessed before (pre) and 25 min after completion of one set (I), five (V) and ten (X) sets of resistance exercise. Biopsies were taken at the same time points and analyzed via western blot for RyR1 phosphorylation. An additional biopsy was taken after ten sets from the non-loaded leg as negative control. (TIF) [file pone.0199307.s004.tif]

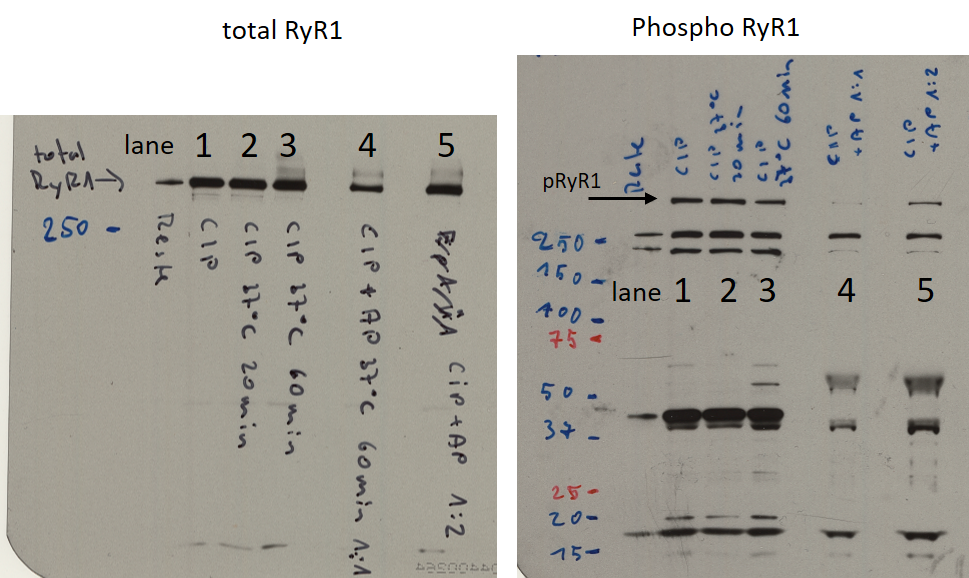

Supplement: S5 Fig — In short: Tissue was prepared as described in the section Methods, Western Blot. Crude cell lysates were incubated with: Line 1: CIP-Buffer, without AP (calf intestinal [CIP]), for testing of possible influence of Buffer itself on pRyR1 and total RyR1 signal. Line 2 and 3: CIP-Buffer without AP, incubated for 30 (line 2) and 60 (line 3)min at 37°C, for testing of possible influence of incubation temperature on pRyR1 and total RyR1 signal. Line 4 and 5: CIP-Buffer with AP added in two different concentrations (recommended → line 4 and lower → line 5). Following incubation with total RyR1 (mouse), a clear signal is visible at predicted band size, which is quite even in intensity across the lines. After stripping the membrane and reincubation with pRyR1 antibody (rabbit), a clear reduction in signal intensity is observable only in the AP added lines 4 and 5. This is a positive indication for us that pRyR1 antibody recognizes differences in phosphorylation and that our results are not based on technical issues. (TIF) [file pone.0199307.s005.tif]
